# Mathematical functions for modeling the variation of specific medical criteria in some infectious diseases

**Published:** 2008-02-25

**Authors:** Streinu-Cercel Adrian, Costoiu Sergiu, Mârza Maria, Aramă Victoria, Streinu-Cercel Anca, Mârza Monica

**Affiliations:** * ”Carol Davila” University of Medicine and Pharmacy, Bucharest, Romania; ** ICPE Bucuresti,-SAPI Dept, Bucharest, Romania

**Keywords:** biomedical engineering, infectious diseases, biotechnology

## Abstract

The paper presents the definition of rules for the medical algorithms in some infectious diseases and also the mathematical functions for modeling: interrogation rules to elaborate specific concepts and work methods in HIV/AIDS infection, interrogation rules to elaborate specifically concepts and work methods in SEPSIS, initial conditions regarding the introduction of the notion of stochastic correlation coefficient, determination of the the stochastic correlation coefficient through the method of reciprocal comparison of the variables, determination of the influential ponders through reciprocal comparison of the characteristics, models for the study of the variation of the specific medical criteria of some infectious diseases in the case of adopting the analysis method of reciprocal non comparison

## 1. Introduction

For the modeling process in medical research to ensure human health by treating infectious diseases we used, for the mathematical, functions, the stochastic correlation coefficient.

In the advanced processing of information, the demands have imposed a quality augmentation of information, which led to the diversification of the general system of synthetic indicators, respectively of the medical characteristics system that provides the health state of a patient at a given time. In this case, a permanent improvement of the indicators' system is necessary because these indicators are used in modeling mathematical processes that correlate the information obtained from the diagnostic made by the specialized medical staff. The demands imposed for the indicators' system are as follows:

- if we consider two groups of indicators then we must establish if there are any influences between the two, in the case of the mathematical model based on the reciprocal comparison of the medical characteristics we can distinguish the subarrays "S1" and "S2".

- for the precedent problem, if we have an affirmative answer, we must establish the sense of this influence, which means that one must establish the determinant group and the determined group

- "S1" stands for the subarray of the medical characteristics of which values have to be as high as possible to improve the health state of the patient;

- "S2" stands for the subarray of the medical characteristics of which values have to be as low as possible to improve the health state of the patient;

- the intensity of the influence of the determinant group over the determined group must be evaluated, for the mathematical model based on the reciprocal comparison of the medical characteristics (medical investigations), the intensity of the determinant group over the determined group is correlated through an influential balance of one medical characteristic over the absolute synthetic indicator of medical correlation that stands for the health state of the patient.

- based upon the information regarding the actual aspect of the dependence between the two groups (subarrays) by defining the relationships between the elements of the two groups (subarrays), we can receive results regarding:

- defining mathematical functions for modeling;

- defining the rules for medical algorithms in some infectious diseases;

- creating the array of semantical terms, from the medical point of view, in some infectious diseases;

- creating the array of the medical characteristics for the analysis in some infectious diseases;

- creating specific database;

- creating computerized solutions related to the realized mathematical model;

- defining the analysis criteria of the results obtained from the achieved software solutions;

- analyzing and formulating the possible extensions for the interpretation of the obtained results

so that in the final state we will provide solutions for solving the prognosis problems, and also the decisional ones of the diagnosis based upon the indicators (medical characteristics) that are a part of both groups, determinant and determined.

## 2. Initial conditions regarding the introduction of the notion of stochastic correlation coefficient

X, Y variables are represented through strings of values

$${\left\{x_{i},y_{i}\right\}}_{i=1,...,n}$$.

If one observes that for some value

$$x_{i0}$$

of the X variable doesn't always correspond to a given value

$$y_{i0}$$

of the Y variable, than the dependence between the variables X and Y will be one of probabilistic type: "

$$\phi \left(x_{i},y_{i}\right)=P\left[\left(Y=y_{i}\right)|\left(X=x_{i}\right)\right]$$

", "

$$\psi \left(x_{i},y_{i}\right)=P{\left[\left(X=x_{i}\right)|\left(Y=y_{i}\right)\right]}_{i=1,2,...,n}$$

" where

$$\phi \left(X_{i},Y_{i}\right)\neq \psi \left(X_{i},Y_{i}\right)$$.

In the case in which the X and Y variables are known all the possible values and also all the corresponding probabilities, the

ϕ

and

ψ

characteristics can be determined using the bidimensional repartition of the random vector (X, Y).

The dependence of probabilistic (stochastic) type between X and Y variables can be characterized based upon two different aspects:

- firstly, if one considers X and Y variables as marginal repartitions of a given bidimensional variable, in which case their dependence is a global one between X and Y of equal intensity in both ways the mathematical description is:

$$\phi \left(X,Y\right)=\rho \left(X,Y\right)=\frac{\mathrm{cov}\left(X,Y\right)}{\sigma \left(X\right)\sigma \left(Y\right)}$$

(1)

with

ρ(X,Y)∈[−1,1],(∀)X,Y

- secondly, the relation between X, Y variables can be characterized also through conditional variables, in which case X and Y variables are considered unconditional variables and the mathematical description is:

$$D^{2}\left(Y\right)=M_{x}\left[D^{2}\left(Y/X=x\right)\right]+D_{x}^{2}\left[M\left(Y/X=x\right)\right]$$

(2) 

A non symmetric measurement of the stochastic dependency of the Y variable rationed to the X variable is achieved by the correlation ratio "

$$\eta^{2}\left(Y,X\right)$$

". Eta is a coefficient of nonlinear association. For linear relationships, eta equals the correlation coefficient (Pearson's r). For nonlinear relationships it is greater -- hence the difference between eta and r is a measure of the extent of nonlinearity of relationship. Just as r² is interpreted as the percent of variance in one variable explained linearly by the other, eta2 is the percent of variance in the dependent variable explained linearly or nonlinearly by the independent variable. This interpretation requires that the dependent variable be interval in level, and the independent variable be categorical (nominal, ordinal, or grouped interval).

$$\eta^{2}\left(Y,X\right)=\frac{D_{x}^{2}\left[M\left(Y/X=x\right)\right]}{D^{2}\left(Y\right)}$$

(3)

$$\eta^{2}\left(Y,X\right)=\frac{D^{2}\left(Y\right)-M_{x}\left[D^{2}\left(Y/X=x\right)\right]}{D^{2}\left(Y\right)}$$

(4)

$$\eta^{2}\left(Y,X\right)=1-\frac{M_{x}\left[D^{2}\left(Y/X=x\right)\right]}{D^{2}\left(Y\right)}$$

(5)

In the same way we use the correlation ratio "

$$\eta^{2}\left(X,Y\right)$$

" for the dependency of the X variable rationed to the Y variable:

$$\eta^{2}\left(X,Y\right)=1-\frac{M_{x}\left[D^{2}\left(X/Y=y\right)\right]}{D^{2}\left(Y\right)}$$

(6)

Generally

$$\eta^{2}\left(X,Y\right)\neq \eta^{2}\left(Y,X\right)$$.

The averages of the conditioned variables

$$\left[\frac{Y}{X}\right]=x$$,

$$\left[\frac{X}{Y}\right]=y$$

are functions of x and respectively of y. The regression function, of the Y variable rationed to the X variable is:

$$f\left(x\right)=M\left(\frac{Y}{X}=x\right)$$

(7)

and the regression function, of the X variable rationed to the Y variable is:

$$g\left(x\right)=M\left(\frac{X}{Y}=y\right)$$

(8)

From the point of view of the analysis of the stochastic dependency of X and Y variables, we mention:

- if the X and Y variables are in a ratio of functional dependency of determinist type, the dependency having the aspect

y=ϕ(x),

then :

$$\eta^{2}\left(X,Y\right)=\eta^{2}\left(Y,X\right)=1$$

(9)

And the regression charts will converge with the chart of the function

$$\eta^{2}\left(Y,X\right)$$

**Figure 1:**
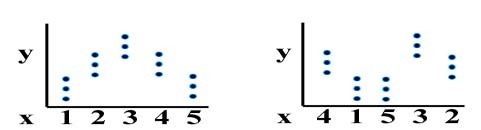
Chart of the function η2 (Y,X)

- if the X and Y variables are in a ratio of functional dependency of linear type, then:

$$\rho^{2}\left(X,Y\right)=1$$

(10)

- in the case of a functional nonlinear dependency, the situation is characterized by the mathematical model associated to the PAIM application, it is possible that

ρ(X,Y)

to take any value from the interval [-1,1].

In the case of the dependency of a random variable in ratio to a group formed from more than just one variable it is important to define the influence of each variable, considered separately as also in a group that has at its turn interdependency relationships.

In the construction of the mathematical model of the PAIM application, the variables dependency in ratio to a group that contains more than just one variable and also the interdependency between them are taken in consideration by the subarrays "S1" and "S2" and by the preference relation between the variables, a relation built by the specialized medical staff.

## 3. Determination of the stochastic correlation coefficient (the ultimate medical synthetic correlation indicator) by the reciprocal comparison method of the variables (medical characteristics)

In the construction of this mathematical model we started by adopting a general function that has the following form:

$$N_{taj}=f\left(K_{ij}\right)=f\left(K_{1j},K_{2j},...,K_{nj}\right)$$

(11)

where 

-

$$K_{ij}$$

represents the variable that takes in account the values of the medical characteristics for each "j" health state; in this way it is concludingly expressed the health state of a person at the "j" moment, that allows the realisation of the biomedical classification;

-

$$N_{taj}$$

represents the stochastic correlation coefficient, in the following, defined as the ultimate medical synthetic correlation indicator of the health state at the "j" moment for the "i" person.

The ultimate medical synthetic correlation indicator of the health state at the "j" moment for the "i" person, "

$$N_{taj}$$

" is quantified by the relation:

$$N_{taj}=a\prod_{i=1}^{m}{\left(\frac{K_{ij}}{K_{il}}\right)}_{\begin{array}{l} i\in S_{1}\\ l\in P \end{array}}^{\gamma_{ij}}\cdot \prod_{i=1}^{n}{\left(\frac{K_{il}}{K_{ij}}\right)}_{\begin{array}{l} i\in S_{2}\\ l\in P \end{array}}^{\gamma_{ij}}$$

(12)

where

-

$$K_{il}$$

represents the absolute value of the homonym characteristic “i” of the referential state “l” respectively the normal health state;

-

$$\gamma_{ij}$$

represents the ponders’ influence of the variable (medical characteristics) “i” over the ultimate medical synthetic correlation indicator of the "j" health state respectively the important coefficients of the “i” characteristics of the “j” state;

- a is a constant for the definition of the reference of the ultimate medical synthetic correlation indicator of the “l” state adopted as a reference state (for better correlation “a=1000”)

- P is the array of the patients whose medical characteristics (medical investigations) are compared.

The correlation between the subarrays S1" and "S2" of the medical characteristics is given by:

$$S_{1}\cup S_{2}=S$$

(13)

where “S” is the array of all medical characteristics (medical investigations) that define the health state of a patient.

Due to the ultimate medical synthetic correlation indicator it becomes possible to track of the dynamics of the health care at an individual level, as the realisation of the classification, from the biomedical point of view, of the compared patients, in ratio with a “referential” patient, which is characterized by the values of the medical investigations that define the normal health state.

For an effective calculus of the ultimate medical synthetic correlation indicator we pass on a logarithmic scale the relation (12) and we obtain:

$$\log N_{taj}=\gamma_{ij}\log\prod_{i=1}^{m}{\left(\frac{K_{ij}}{K_{il}}\right)}_{\begin{array}{l} i\in S_{1}\\ l\in P \end{array}}^{}+\gamma_{ij}\log\prod_{i=1}^{n}{\left(\frac{K_{il}}{K_{ij}}\right)}_{\begin{array}{l} i\in S_{2}\\ l\in P \end{array}}^{}+\log1000$$

(14)

With the (14)th relation it is analytically formalized the mathematical model which realizes that: the creation of biomedical specific classification, the opportunity creation of the monitorization for the anti-microbian drugs, the evaluation of the diagnostic prognosis, the evaluation of the health state evolution of the patient.

An important remark is that

$$K_{ij}$$

is reported to the same type of medical investigations, which allows working with different factors of different influential senses. In this way the normal health state of a patient is described and also its approach to the effective state of the investigated patient.

From the mathematical point of view, the built relation indicates the following interdependencies between the ultimate medical synthetic correlation indicator and medical investigations that characterize the health state:

-

$$N_{taj}$$

is directly proportional to the values of the medical characteristics that have to be as high as possible, by comparison to constant medical characteristics “

$$K_{il}$$”

that define the normal from the medical point of view

-

$$N_{taj}$$

is inversely proportional to the values of the medical characteristics that have to be as low as possible, by comparison to constant medical characteristics “

$$K_{il}$$”

that define the normal from the medical point of view

The variables dependency in ratio to a group that contains more than just one variable and also the interdependency between them are taken in consideration by the subarrays "S1" and "S2", which imposed that the influential ponders “

$$\gamma_{ij}$$”,

considered in the anterior relations have to be broken in two groups as the medical characteristics must be, some as high as possible “

$$i\in S_{1}$$”

and others as low as possible “

$$i\in S_{2}$$”.

## 4. Determining the influential ponders by reciprocal comparison of the characteristics

To determine the exponents of “

$$\left(\frac{K_{ij}}{K_{il}}\right)$$”

and “

$$\left(\frac{K_{il}}{K_{ij}}\right)$$”

respectively of the values for the influential ponders of the “i” characteristics over the ultimate medical synthetic correlation indicator of the "j" state, also the importance coefficients of the “i” characteristics at the "j" state,

$$\gamma_{ij}$$

we use the procedure STEP METHOD (STEM) that presumes the determining of the influential ponders by reciprocal comparison of the characteristics.

The adaptation of the STEM method for determining the influential ponders of the “m” characteristics is made by the hypothesis that in the reciprocal comparison, two by two, of the medical characteristics that define the health state are known the important relations between them. So, no matter the two characteristics “Ki” and “Kj”, by comparing themselves we can say if they are as equally important or which is the most important. Following this pattern, to the “K1”, “K2”,…,”Km” we can attach a quadratic matrix,

$$A=\left\|a_{ij}\right\|$$,

i,j=1, 2,…, n of whose terms are defined as:

$$A=\left\|a_{ij}\right\|=\{\begin{array}{cccc} 1 & K_{i} & I & K_{j}\\ 2 & K_{i} & P & K_{j}\\ 4 & K_{i} & PP & K_{j}\\ 0 & K_{i} & N & K_{j} \end{array}$$

(15)

where:

- I admits that the “Ki” characteristic is as equally important as the “Kj” characteristic

- P admits that the “Ki” characteristic is more important than the “Kj” characteristic

- PP admits that the “Ki” characteristic is much more important than the “Kj” characteristic

- N expresses all the other cases.

The common influential ponders “

$$\gamma_{ij}$$”

for the “i” characteristic of all investigated states is defined as:

$$\gamma_{ij}=\frac{\sum_{i=1}^{m}a_{ij}}{\sum_{i=1}^{m}\sum_{j=1}^{m}a_{ij}}$$

(16)

where

$${\left\|a_{ij}\right\|}_{i,j=1,2,...,n}$$

are elements of the quadratic matrix “A”.

## 5. The array for the medical correlation indicators

The elements of the array for the semantic terms "Ssem" have different influential senses. Starting from the elements of this array, through a conceptual modeling process one forms the array of the correlation indicators, which allowed the construction of the quantification algorithms of these concepts.

With the elements of the "Ssem" array we can quantify a level of the correlation indicators that determine a "value" that is compared to a "referential value", which characterize the normal state from the medical point of view. Now we are able to give a global expression for the complete anamnesis of health state evaluation: present symptoms, the apparition moment of the present symptoms, the dynamics of the present symptoms, physiological precedents, vaccination precedents, pathological precedents, previous treatments, present treatments, risk factors, the existence of the conditions that provide the apparition of opportunistic infections with different influential senses.

## 6. Mathematical functions for modeling the variation of the specific medical criteria of some infectious diseases in the case of adopting the methods of the variable reciprocal non comparison analysis 

The medical information processing the transfer of the research results in treating some infectious diseases supposes the interpretation of the given results by medical criteria (medical investigation) specific to some infectious diseases. The interpretation of results can be done, firstly, by applying different categories of functions, in ratio to intervals of medical normality of these criteria. Because the intervals of medical normality are intervals of real number, for the modeling we will adopt real functions of real variables.

We choose functions then we linked them to medical criteria: polynomial 6th degree function – leukocyte number, logarithmic function – fibrinogen, exponential function – albumin, power function – creatinine studied for a number of 16 patients.

**Figure 2:**
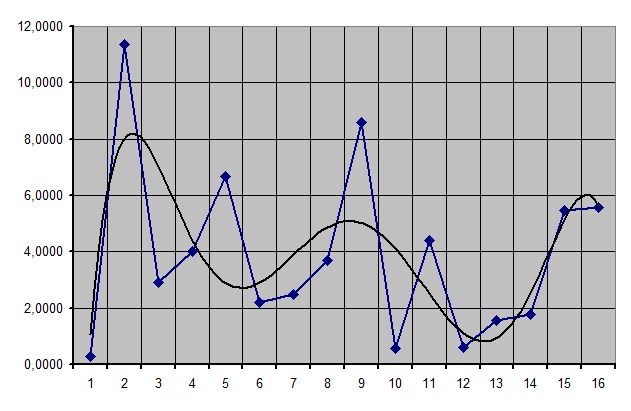
Results after treating the leukocyte number function

## 7. Acknowledgments 

1. Ministry of education, research and youth;

2. Medical Scientific Academy of Romania.

3. VIASAN PROGRAMME.
